# Acute appendicitis complicating pregnancy: a 33 case series, diagnosis and management, features, maternal and neonatal outcomes

**DOI:** 10.11604/pamj.2018.30.212.14515

**Published:** 2018-07-16

**Authors:** Mohamed Amine El Ghali, Ons Kaabia, Zaineb Ben Mefteh, Maha Jgham, Amel Tej, Asma Sghayer, Amine Gouidar, Afra Brahim, Rafik Ghrissi, Rached Letaief

**Affiliations:** 1 Department of General and Digestive Surgery Farhat Hached Universitary Hospital, Sousse, Tunisia; 2 Department of Gynecology and Obstetrics, Farhat Hached Universitary Hospital, Sousse, Tunisia; 3 Department of Anaesthesia, Farhat Hached Universitary Hospital, Sousse, Tunisia; 4 Department of Pediatric, Farhat Hached Universitary Hospital, Sousse, Tunisia

**Keywords:** Appendicitis, regnancy, diagnosis, surgery, mater-nofetal prognosis

## Abstract

The occurrence of acute appendicitis during pregnancy may pose diagnostic and therapeutic difficulties. In fact pregnancy can make the clinical diagnosis delicate and the use of morphological examinations is still subject to controversy. The debates concerning the ideal surgical approach during pregnancy continue. On the other hand, in some cases the occurrence of acute appendicitis, especially in its complicated form, which is frequent in pregnant women, exposes to obstetrical complications and an increased risk of premature delivery We aims to describe the clinical and management features of acute appendicitis in pregnant women and the maternal and neonatal outcomes and carry out a review of the literature on this topic. It is a retrospective analysis of a series of 33 cases of appendicitis in pregnant women who were diagnosed and managed, in collaboration between the departments of General and digestive surgery, Gynecology and Obstetrics and Anaesthesia at Farhat Hached Universitary Hospital Sousse Tunisia between January 2005 and December 2015. The average age of the patients was 29 (20-40). Fourteen patients were in the first trimester, twelve in the 2^nd^ and seven in the third trimester. The main symptom was pain in the right iliac fossa. The mean delay between consultation and surgery was 2.7 days. Twenty five patients had a preoperative ultrasound. Eight of the 33 pregnant patients presented complicated appendicitis with localized or generalized peritonitis. Thirty patients underwent laparotomic appendectomy: 28 with a Mc Burney incision and 2 with a midline incision and only three patients underwent laparoscopy. Preventive tocolysis was given to 14 patients, maternal mortality was null. Twenty four pregnancies were followed until delivery: one case of premature birth and one case of preterm labor were observed. Pregnancy makes it difficult to diagnose appendicitis, which explains the high rate of complicated acute appendicitis in our series. An early treatment improves maternal and fetal outcome.

## Introduction

Acute appendicitis is the most common non-obstetrical surgical emergency during pregnancy [1]. Its incidence in pregnant women varies from 50/100 000 to 130/100 000 [2]. The association of this surgical emergency and pregnancy is a serious one that involves the maternal and fetal prognoses. Symptoms are polymorphous and misleading, causing misdiagnosis and/or diagnostic and therapeutic delay [3]. The objectives of this study were to describe the clinical, para-clinical and therapeutic features of acute appendicitis in pregnant women and the factors influencing materno-fetal outcomes.

## Methods

It is a descriptive study with a retrospective analysis of a series of 33 cases of appendicitis in pregnant women who were diagnosed and managed, in collaboration between the departments of General and digestive surgery, Gynecology and Obstetrics and Anaesthesia at Farhat Hached Universitary Hospital Sousse Tunisia between January 2005 and December 2015. The information was collected from the files and charts of the patients in the three different departments. In comparison with the physiological hyperleukocytosis in pregnant women, leukocytosis was considered to have a white blood cell count> 12000 cells/mm^3^. The data was analyzed using SPSS 20.

## Results

**Figure 1:**
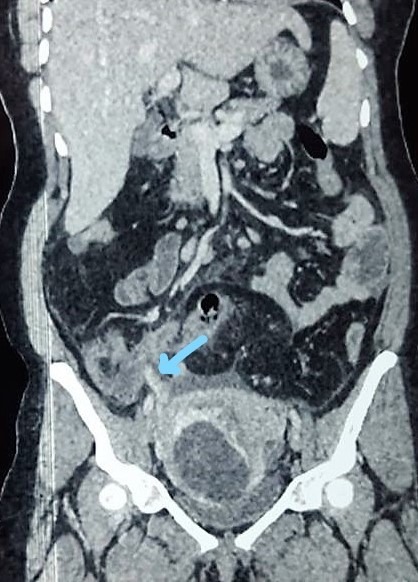
Coronal reconstruction showing complicated acute appendicitis and pregnant uterus

**Figure 2:**
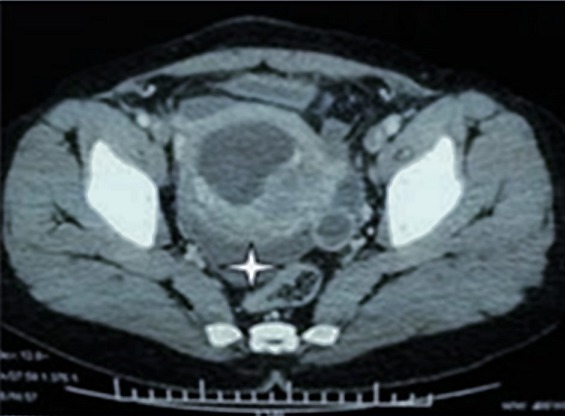
Abdominal CT showing accute appendicitis during pregency Douglas abces complicating acute appendicitis

Of the 2982 patients who underwent surgery for acute appendicitis during the study period at the department of General and digestive surgery at Farhat Hached Universitary Hospital Sousse Tunisia, 33 cases of acute appendicitis were observed in pregnant women; leading to a prevalence in the general population of 1.1%. The mean age of our patients was 29 years, with extremes ranging from 20 to 40 years. The diagnosis of acute appendicitis was observed in the first trimester of pregnancies in 14 patients, which is twice the number of cases observed in the 3rd trimester (n = 7) (Table 1) In one case the diagnosis of acute appendicitis complicated with generalized peritonitis was only made during an emergency caesarian section for a suspicion of acute fetal distress. Abdominal pain was the main symptom and the first symptom to appear in all patients. It was mainly located in the right iliac fossa (RIF) (78.8 % of cases) Vomiting was an associated symptom in 69.7% (Table 1). The major sign during the abdominal palpation was a sensitivity of the right iliac fossa. Moreover, the sign of Rovsing was positive in 6 patients (18.2 %). Abdominal ultrasound was performed in 25 patients (75.7% of cases). The diagnosis of acute appendicitis was made on the ultrasound findings in 11 patients (44.0%). The sensitivity of this exam is 44.0% and the Positive Predictive Value is 100%. All the pregnant patients in whom the appendix was not seen during the abdominal ultrasound were in the 3^rd^ trimester of their pregnancy. Abdominal CT was requested for 2 patients in the 3^rd^ trimester (Figure 1). In one patient, it showed an appearance of acute appendicitis complicated by peritonitis with multiple intraperitoneal collections (Figure 2). Concerning the delay between consultation and surgery: 22 patients were operated on the same day of admission, 8 the next day and three patients 2 days later.

**Table 1:** Clinical symptomatology by term of pregnancy

Terme	Seat of Pain		temperature		vomiting	diarrhea	Urinair signs
T1=14	Right iliac fossa	11	37°C	6	10	0	0
	Generalized	2	37,5-38°C	6			
	Péri ombilical	1	>38°C	2			
T2=12	Right iliac fossa	10	37°C	6	9	1	3
	Generalized	1	37,5-38°C	5			
	Peri- ombilical	1	>38°C	1			
T3=7	Right iliac fosse	5	37°C	1	4	0	3
	Généralizaied	1	37,5-38°C	2			
	Péri omoblical	1	>38°C	4			

**Figure 3:**
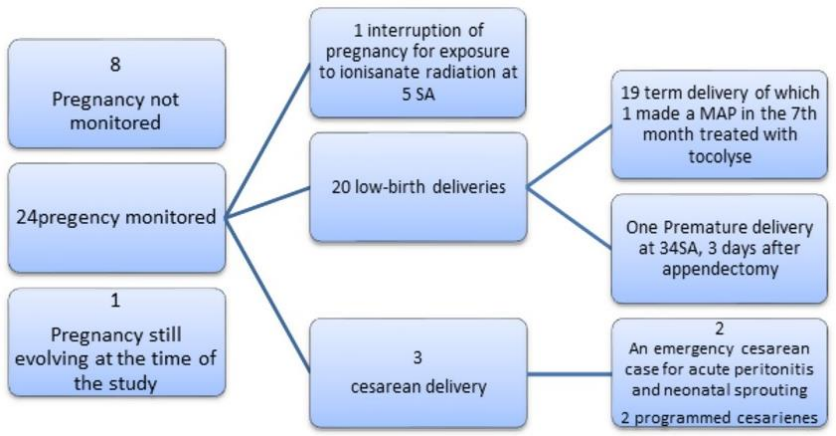
Obstetrical outcomes

A pre-operative antibiotic therapy based on Beta-lactamin and Metronidazole was established in these 2 patients. 76.0% of our patients received intra operative antibiotic therapy with Amoxicillin +Clavulanic Acid, during the induction of anesthesia. Eight patients underwent post-operative antibiotic therapy based on Beta-lactamin and Metronidazole during 10 days. During the surgery, 30 patients had a laparotomy and 3 patients had a laparoscopy (Table 2). All three laparoscopies were in patients between 12 and 18 WA. In all three patients, the acute appendicitis was uncomplicated .The mean duration of the operation was 54 minutes, with extremes ranging from 30 minutes to 130 minutes. Surgical exploration showed uncomplicated appendicitis in 25 cases and complicated in 8 cases (Table 3). The appendix was in the lateral-internal caecal position in 78.8% of the cases and retro caecal in 21.2% of the cases.The histological examination of the appendix was in most cases catarrhal, suppurative, phlegmonous and rarely gangrenous. The mean hospital postoperative stay was 2.7 days with extremes ranging from 1 to 5 days. Maternal and fetal outcomes were studied in 24 patients (Figure 3). Fourteen patients had a perioperative preventive systemic tocolysis (Table 4) (intramuscular and vaginal progesterone before 22 WA and oral Calcium channels Inhibitors between 22 and 35 WA. Fetal heart sounds were present and regular in all patients. There was a threat of perioperative preterm delivery in a two patients: one in a pregnant patient at 28 WA (weeks of amennorrhea) with a good evolution after intravenous tocolysis (Calcium channels Inhibitors) and one in another pregnant patient resulting in a premature delivery at 34WA + 5days (ie, 3 days after surgery) despite the same protocole of intravenous tocolysis. Maternal mortality was nil but maternal morbidity was not: in fact 2 cases of pituitary apoptosis complicating severe maternal hemorrhage were noticed in association with a case of prolonged paralytic ileus in a patient operated for generalized peritonitis.

**Table 2:** Surgical approach during pregnancy

	T1	T2	T3	Total
Laparoscopy	1	2	0	3
Mac Burney's Incision	10	9	6	26
Median Incision	0	1	1	2
Modified Mac Burney Incision	2	0	0	2

**Table 3:** Operative finding

Uncomplicated appendicitis		25
Complicated appendicitis	Apendicular abcess	1
	Localized peritonitis	3
	peritonitis	4

**Table 4:** Indications of tocolysis after acute appendicitis diagnosis

Preventive	12
Curative	2
Pre-operative	1
Post-operative	1

## Discussion

Acute appendicitis is the most common non-obstetrical surgical emergency during pregnancy [1,2]. It accounted for 65.6% of non-traumatic digestive emergencies in pregnant women, with a general prevalence of 0.1 to 0.2% [1]. It is higher in our series with 1.1%. Andersson et al found an inversely proportional relationship between appendicitis and pregnancy and it was mostly in the 3rd trimester [4], unlike our own findings were most patients were diagnosed during the 1^st^ and 2^nd^ trimester of pregnancy. Andersson et al concluded that pregnancy protects against appendicitis [4]. However, other studies, like ours, have shown that the association of appendicitis and pregnancy is more frequent during the 1^st^ and 2^nd^ trimester [5] and rare in the last weeks of pregnancy [6]. And a recent review of the literature found an incidence of acute appendicitis slightly elevated in the 2^nd^ trimester of pregnancy [5], which is concordant with our results (42.4% of our patients were in their first trimester, which was twice as many as in the third trimester). The clinical and radiological diagnosis of acute appendicitis during pregnancy is challenging [7]. The pros and cons of this diagnosis must be weighed because excessive intraoperative pelvic manipulations increase the risk of threat of premature delivery and neglected appendicitis exposes to the risk of serious materno-fetal complications. In the first trimester, the semiology of appendicitis is no different from that observed in non-pregnant [8]. Abdominal pain is the most constant sign. Since 1932, Baer et al. demonstrated that the appendix and the caecum moved during the pregnancy progressively and laterally in a counter-clockwise direction outside the pelvis [9]. Subsequently, this theory was called into question. In fact, Mourad et al [10] found that in the majority of pregnant women, pain was located in the RIF and this is regardless of the term of pregnancy. The same results were confirmed by the studies of Yilmaz and Melnick [11] and our own results: RIF was the predominant site of pain (78.8% of our patients) regardless of the term of pregnancy. Other functional signs are common during pregnancy, which deprives them of their diagnostic interest [1]. As with all acute appendicitis, fever is not constant or specific [11,12]. Therefore its presence most often in favor of a complicated appendicitis [2].

Of eight patients in our series with a fever, seven had a complicated acute appendicitis. The higher incidence of complicated appendicitis in pregnant women is not only due to the delay in diagnosis and/or in management but also to some patho-physiological changes. In fact, pregnancy puts the woman in a state of relative immune suppression that alters the normal inflammatory response. On the other side, pregnancy may result in a hyper-vascularization of the appendix that promotes early lymphatic dissemination, and uterine contractions hamper peripheral appendicular adherence. Moreover, pregnancy makes the abdominal palpation signs difficult to appreciate given the increase in the uterine volume [8]. As the pregnancy progresses, abdominal sensation and defense are less evident because of the laxity of the abdominal wall and the increase in the space between the abdominal wall and the appendix [12-14]. The abdominal contracture, master symptom in case of appendicular peritonitis is often inconstant. The uterine contraction rapidly evolving in contracture causes suspicion of an obstetrical complication and may lead to premature delivery [15]. The patient that had a Caesarean section for suspicion of acute fetal suffering in our series illustrates this notion. "The American College of Radiologists (ACR) Appropriateness Criteria 2011" considered abdominal ultrasound, the initial imaging of choice, in front of any suspicion of acute appendicitis in pregnant women [16]. It is also of interest to eliminate an associated adnexal or obstetrical pathology, to document the pregnancy by specifying the age gestation and the fetal vitality. However, the false negative rate by ultrasound that is variable in the literature puts these recommendations into question [17,18]. Our study as well as several recent studies have also shown a low ultrasound sensitivity varying from 36 to 46% [17,18]. These results are explained according to some authors, by the fact that the ultrasounds are "operator dependent". The pregnant uterus, in our cases, impeded the visualization of the appendix in the 3^rd^ trimester and some authors suggest that abdominal CT can be of help. Indeed, it has a sensitivity ranging from 77% to 98% and a specificity of 83% to 100% in the diagnosis of acute appendicitis [19,20]. Its superiority to abdominal ultrasound is proved by a large meta-analysis [21]. The strong reluctance to use CT during pregnancy is mainly due to the teratogenic risks of ionizing radiation especially during the organogenesis period. The committee of the Society of magnetic resonance imaging recommends MRI for pregnant women [21]. A recent study showed a high rate of MRI visualization of the appendix, with a sensitivity of 91.7% and a specificity of 95.3%. Indeed, it is capable of eliminating the diagnosis of acute appendicitis in pregnant women [22] and consequently eliminates the need for unnecessary appendectomies following inconclusive imaging [17, 22, 23]. The limited access to MRI in our hospital constitutes a limiting factor that explains the absence of MRI in our study.

The gold standard treatment of acute appendicitis in pregnant women is a surgical appendectomy. A recent meta-analysis compared the efficacy of curative antibiotic therapy to an appendectomy in patients with uncomplicated acute appendicitis. This meta-analysis concluded that antibiotic treatment would be effective and could be used as a first line management option for uncomplicated appendicitis. The disadvantage of this conservative attitude is the risk of recurrence (30% less than 1 year) and the frequency of complicated forms [24]. Abbasi et al [25] found that pregnant women receiving conservative treatment had a higher rate of septic shock, peritonitis and thromboembolic events compared to surgically treated women. This non-operative attitude cannot therefore be advocated in pregnant women with acute appendicitis. The management of acute appendicitis in pregnant women should be multidisciplinary, with the presence of the surgeon, anesthesiologist, obstetrician and possibly the pediatrician in order to minimize the risk of morbidity and maternal-fetal mortality [14]. Laparoscopy has gradually gained universal approval for the treatment of acute appendicitis, is associated with decreased postoperative pain and decreases the risk of uterine irritability [26], decreases maternal postoperative hypoventilation, fetal hypoxemia, it also reduces the rate of spontaneous abortions, the threat of premature delivery and premature birth [26]. Several studies have concluded that the use of laparoscopy during pregnancy is safe for both the mother and the fetus [14, 25]. Laparoscopy in the 1^st^ and 2^nd^ trimester of pregnancy has a dual interest, both diagnostic and therapeutic [26]. However, some authors consider that the laparoscopic approach is possible also in the third trimester by exploiting the free space between the uterine fundus previously identified and the xiphoid appendix [26]. During laparotomy, the manipulation of the gravide uterus increases the risk of premature delivery, whereas the laparoscopic approach is less invasive and reduces the risk of appendectomy and consequently the risk of fetal loss [27]. But a recent systematic review and meta-analysis comparing appendectomy by laparoscopy and laparotomy during pregnancy, provided with a poor quality evidence that laparoscopy in pregnant women may be associated with an increased risk of fetal loss [28]. Subsequently, a consensus conference developing Guidelines for laparoscopic appendectomy concluded that there is no consensus regarding laparoscopic appendectomy during pregnancy, although this technique is considered safe for materno-fetal prognosis especially in the second trimester. However, level IIb evidence is not considered sufficient to consider laparoscopic appendectomy as the gold standard treatment in pregnant women [29]. Our series showed that mac-Burney incision in safe and sure in all therm of pregency.

Surgical complications may occur in the form of deep peritoneal abscess, or parietal suppuration, which remains the most frequent [1] and its frequency ranged from 8% to 15% [11]. In our study no peri operative septic complications have been reported. Peri-operative antibiotic therapy aims to reduce the risk of post-operative septic complications. This antibiotic therapy should be a broad-spectrum one covering anaerobic germs as well as Gram-negative ones [29]. The prevent and treat premature delivery is essential because it is the most threatening obstetrical complication. It is due on the one hand to the release of the factors of inflammation and on the other hand to the mechanical manipulation of the uterus during the procedure for appendectomy. According to Lebeau, tocolysis should be systematically prescribed in all patients to prevent uterine contractions and therefore no cases of abortion or threatened preterm labor have been reported [1]. Other authors consider that the indication of this tocolysis is mandatory only in the period from the end of the first trimester to the 34^th^WA [15]. The maternal and fetal prognosis depends on the severity of the disease and the therapeutic delay Several studies have found that maternal-fetal morbidity and mortality (abortion, threat of abortion, premature delivery, or threat of preterm delivery) increase in case of appendix perforation or in case of late term [1,25,30]. Other studies have not found any correlation between these parameters and the occurrence of obstetrical complications [2]. In our series, there was only two cases of threat of premature delivery whom one was managed with tocolytics and only one case of preterm delivery at 34 WA + 5days despite appendectomy and tocolysis. Fetal mortality varies in the literature. It is mainly related to perinatal infection and to prematurity (29), Several series [4,7, 30] reported an increase in fetal mortality in cases of appendix perforation or peritonitis, with a rate rising from 1.5% to 20-35% in the case of peritonitis [12]. Fetal morbidity is marked mainly by prematurity that occurs during any abdominal surgery during pregnancy. Some studies have shown an increased risk of prematurity in the week after appendectomy [1,3]. Concerning the delivery, a caesarean section in a septic environment exposes to the infection of the uterine wound, to endometritis and to the risk of opening of the hysteroraphy [28]. Moreover, the uterine scar transforms subsequent pregnancies into pregnancies at high risk of uterine rupture. A caesarean section should therefore be performed only for obstetrical indications. In our series we had a case of acute fetal suffering revealing a generalized appendicular peritonitis misdiagnosed and only discovered at the time of the Caesarean section. The appendectomy was performed during the same surgery.

## Conclusion

Acute appendicitis in pregnant women is a rare and relatively difficult to diagnose condition. Its clinical polymorphism may lead to a diagnostic delay explaining the high rate of complicated acute appendicitis. Abdominal ultrasound is becoming more and more necessary in the diagnosis process. Laparotomy still finds its place especially in case of emergency. The site of incision could be guided by the seat of the pain. The maternal and especially fetal morbidity and mortality are not to be neglected and premature delivery has to be prevented.

### What is known about this topic

- Acute appendicitis of the pregnant woman poses diagnostic difficulties and is restable due to the long delay for the diagnosis of surgical, obstetrical and sometimes neonatal complication;
- The surgical approach is debated by lapartotomy the site of the incision is controversial. Laparoscopy is a good alternative depending on the obstetric term. In the context of emergency surgery, it is still not available in developing countries.

### What this study adds

- The incision of mac-burney is possible no matter the term of the pregnancy and in case of complicated appendicte;
- Surgical complications, obsterical and neonatal outcome are satisfactory.

## Competing interests

The authors declare no competing interests.

## References

[1] Lebeau R, Diané B, koffi E, Bohoasou E, kouamé A, Doumbia Y (2005). Appendicite aigue et grossessse: à propos de 21 cas. J Gynecol Obstet Biol Reprod.

[2] Andersen B, Nielsen TF (1999). Appendicitis in pregnancy: diagnosis, management and complications. Acta Obstet Gynecol Scand.

[3] Mazze RI, Kallen B (1991). Appendectomy during pregnancy: a Swedish registry study of 778 cases. ObstetGynecol.

[4] Andersson RE, Lambe M (2001). Incidence of appendicitis during pregnancy. Int J Epidemiol.

[5] Franca Neto AH (2015). Acute appendicitis in pregnancy: literature review. Rev Assoc Med Bras.

[6] Al Qudah MS, Amr M, Sroujieh A, Issa A (1999). Appendectomy inpregnancy: the experience of a university hospital. JobstetGynecol.

[7] Tatli F, Yucel Y, Gozeneli O, Dirican A, Uzunkoy A (2017). The Alvarado Score is accurate in pregnancy: a retrospective case-control study. Eur J Trauma Emerg Surg.

[8] Miloudi N, Brahem M, Ben Abid S, Mzoughi Z (2012). Acute appendicitis in pregnancy: specific features of diagnosis and treatment. J Visc Surg.

[9] Hodjati H, Kazerooni T (2003). Location of the appendix in the gravid patient: a re-evaluation of established concept. Int J Gynecolobstet.

[10] Mourad J, Elliott JP, Erickson L, Lisboa L (2000). Appendicitis in pregnancy: New information that contradicts long held clinical beliefs. Am J Obstet Gynecol.

[11] Yilmaz HG, Akgun Y, Bac B, Celik Y (2007). Acute appendicitis in pregnancy-risk factors associated with principal outcomes: a case control study. Int J Surg.

[12] Brown JJS, Wilson C, Coleman S, Joypaul BV (2009). Appendicitis in pregnancy: an ongoing diagnostic dilemma. Colorectal Dis.

[13] Augustin G, Majerovic M (2007). Non-obstetrical acute abdomen during pregnancy. Eur J Obstet Gynecol Reprod Biol.

[14] Chawla S, Vardhan S, Jog B (2003). Appendicitis during pregnancy. Med J Armed Forces India.

[15] Mohsine R, Ismael F, Lekhal B (1996). Péritonite et grossesse. Médecine du Maghreb.

[16] Rosen MP, Ding A, Blake MA (2011). ACR appropriateness criteria right lower quadrant paIn: suspected appendicitis. J Am Coll Radiol.

[17] Israel GM, Malguria N, McCarthy S, Copel J, Weinreb J (2008). MRI vs. ultrasound for suspected appendicitis during pregnancy. J Magn Reson Imaging.

[18] Pedrosa I, Lafornara M, Pandharipande PV, Goldsmith JD, Rofsky NM (2009). Pregnant patients suspected of having acute appendicitis: effect of MR imaging on negative laparotomy rate and appendiceal perforation rate. Radiology.

[19] Freeland M, King E (2009). Diagnosis of appendicitis in pregnancy. Am J Surg.

[20] Van Randen A, Bipat S, Zwinderman AH (2008). Acute appendicitis: meta-analysis of diagnostic performance of CT and graded compression. US related to prevalence of disease. Radiology.

[21] McGory ML, Zingmond DS, Tillou A, Hiatt JR, Ko CY, Cryer HM (2007). Negative appendectmomy in pregnant women is associated with a substantial risk of fetal loss. J Am CollSurg.

[22] Pedrosa I, Levine D, Eyvazzadeh AD, Siewert B, Ngo L, Rofsky NM (2006). MR imaging evaluation of acute appendicitis in pregnancy. Radiology.

[23] Oto A, Ernst RD, Shah R (2005). Right-lower-quadrant pain and suspected appendicitis in pregnant women: evaluation with MR imaging-initial experience. J Radiology.

[24] Varadhan KK, Neal KR, Lobo DN (2012). Safety and efficacy of antibiotics compared with appendectomy for treatment ofuncomplicated acute appendicitis: meta-analysis of randomised controlled trials. BMJ.

[25] Abbasi N, Patenaude V, Abenhaim HA (2014). Management and outcomes of acute appendicitis in pregnancy-population-based study of over 7000 cases. BJOG.

[26] Donkervoort SC, Boerma D (2011). Suspicion of acute appendicitis in the third trimester of pregnancy: pros and cons of a laparoscopic procedure. JSLS.

[27] Cohen-Kerem R, Railton C, Oren D, Lishner M, Koren G (2005). Pregnancy outcome following non-obstetric surgical intervention. Am J Surg.

[28] Wilasrusmee C, Sukrat B, McEvoy M (2012). Systematic review and meta-analysis of safety of laparoscopic versus open appendicectomy for suspected appendicitis in pregnancy. Br J Surg.

[29] Vettoretto N, Gobbi S, Corradi A (2011). Consensus conference on laparoscopic appendectomy: development of guidelines. Colorectal Dis.

[30] Kennedy A (2000). Assessment of acute abdominal pain en the pregnant patient. Semin Ultrasound CT MR.

